# Balanced impacts of fitness and drug pressure on the evolution of PfMDR1 polymorphisms in *Plasmodium falciparum*

**DOI:** 10.1186/s12936-021-03823-x

**Published:** 2021-06-30

**Authors:** Marvin Duvalsaint, Melissa D. Conrad, Stephen Tukwasibwe, Patrick K. Tumwebaze, Jennifer Legac, Roland A. Cooper, Philip J. Rosenthal

**Affiliations:** 1 Department of Medicine, University of California, San Francisco, CA USA; 2 Infectious Diseases Research Collaboration, Kampala, Uganda; 3 Dominican University of California, San Rafael, CA USA

**Keywords:** Malaria, *Plasmodium falciparum*, Drug resistance, Fitness, PfMDR1

## Abstract

**Background:**

Anti-malarial drug resistance may be limited by decreased fitness in resistant parasites. Important contributors to resistance are mutations in the *Plasmodium falciparum* putative drug transporter PfMDR1.

**Methods:**

Impacts on in vitro fitness of two common PfMDR1 polymorphisms, N86Y, which is associated with sensitivity to multiple drugs, and Y184F, which has no clear impact on drug sensitivity, were evaluated to study associations between resistance mediators and parasite fitness, measured as relative growth in competitive culture experiments. NF10 *P. falciparum* lines engineered to represent all PfMDR1 N86Y and Y184F haplotypes were co-cultured for 40 days, and the genetic make-up of the cultures was characterized every 4 days by pyrosequencing. The impacts of culture with anti-malarials on the growth of different haplotypes were also assessed. Lastly, the engineering of *P. falciparum* containing another common polymorphism, PfMDR1 D1246Y, was attempted.

**Results:**

Co-culture results were as follows. With wild type (WT) Y184 fixed (N86/Y184 vs. 86Y/Y184), parasites WT and mutant at 86 were at equilibrium. With mutant 184 F fixed (N86/184F vs. 86Y/184F), mutants at 86 overgrew WT. With WT N86 fixed (N86/Y184 vs. N86/184F), WT at 184 overgrew mutants. With mutant 86Y fixed (86Y/Y184 vs. 86Y/184F), WT and mutant at 86 were at equilibrium. Parasites with the double WT were in equilibrium with the double mutant, but 86Y/Y184 overgrew N86/184F. Overall, WT N86/mutant 184F parasites were less fit than parasites with all other haplotypes. Parasites engineered for another mutation, PfMDR1 1246Y, were unstable in culture, with reversion to WT over time. Thus, the N86 WT is stable when accompanied by the Y184 WT, but incurs a fitness cost when accompanied by mutant 184F. Culturing in the presence of chloroquine favored 86Y mutant parasites and in the presence of lumefantrine favored N86 WT parasites; piperaquine had minimal impact.

**Conclusions:**

These results are consistent with those for Ugandan field isolates, suggest reasons for varied haplotypes, and highlight the interplay between drug pressure and fitness that is guiding the evolution of resistance-mediating haplotypes in *P. falciparum.*

## Background

Malaria remains an enormous problem in tropical regions despite extensive efforts to control and eliminate the disease [1]. Malaria is particularly devastating in Africa, where the vast majority of cases are due to *Plasmodium falciparum*, the most virulent human malaria parasite [2]. With development of resistance to older drugs, standard therapy for falciparum malaria in Africa moved to artemisinin-based combination therapy (ACT) early this century [3]. ACT, including artemether-lumefantrine, artesunate-amodiaquine, dihydroartemisinin-piperaquine, artesunate-mefloquine, and artesunate-pyronaridine, combines a potent and fast-acting artemisinin derivative with a slower acting partner drug [4]. Partner drugs play a key role in assuring the efficacy of ACT, by eliminating remaining parasites after the short acting artemisinins are cleared, and by protecting against emergence of artemisinin resistance. However, resistance to artemisinins in southeast Asia [5] and varied activity of most partner drugs [6] threatens the efficacy of ACT. Interestingly, widely used partner drugs have opposing drug sensitivity profiles, with decreased sensitivity to amodiaquine (a close relative of chloroquine) associated with increased sensitivity to lumefantrine and mefloquine [7].

Varied sensitivities to some ACT partner drugs correlate with polymorphisms in putative drug transporters [8]. The 76T mutation in the *P. falciparum* chloroquine resistance transporter (PfCRT) is associated with resistance to chloroquine and amodiaquine [9, 10], and the K76 wild type is associated with decreased sensitivity to lumefantrine and mefloquine [10]. The PfCRT 76T mutation was previously widespread in Africa [11], but prevalence has decreased with replacement of chloroquine by artemether-lumefantrine as the first-line anti-malarial. Mutations in a second putative drug transporter, the ATP-binding cassette protein PfMDR1, also impact on drug sensitivity [12, 13]. In particular, PfMDR1 N86Y has the same pattern of association as PfCRT K76T, with the 86Y mutation associated with decreased sensitivity to chloroquine and amodiaquine [10, 14], and selected by therapy with amodiaquine [15–17], and the N86 wild type associated with decreased sensitivity to lumefantrine and mefloquine [10, 14], and selected by therapy with lumefantrine [16–19]. As seen for PfCRT 76T, the prevalence of PfMDR1 86Y has decreased markedly in many regions in recent years [11]. Two other common PfMDR1 mutations in Africa, 184F and 1246Y, have uncertain associations with drug sensitivity, although 1246Y was selected by therapy with artesunate-amodiaquine [15] and associated with decreases in both amodiaquine sensitivity and fitness in vitro [20]. Increased *pfmdr1* copy number is associated with decreased sensitivity to mefloquine and lumefantrine [21, 22], but increased copy number is uncommonly seen in African parasites [23–25].

Changes in the prevalence of key transporter mutations with changes in drug utilization suggest a complex interplay between mediators of drug sensitivity and parasite fitness in determining genotypes now circulating in Africa. However, the impacts of PfMDR1 haplotypes on *P. falciparum* fitness are uncertain. In co-culture experiments, wild type parasites outgrew parasites with four introduced PfMDR1 mutations (184F, 1034C, 1042D, 1246Y) [26] and with amplification of *pfmdr1* [27]. However, these experiments did not consider the PfMDR1 86Y mutation, which appears to be the key drug sensitivity mediator for this protein in Africa. In a study in the Gambia before the advent of ACT, the prevalence of PfMDR1 N86 wild type parasites was greater at the beginning of the transmission season than later in that season, consistent with a selective advantage for the wild type parasites when selective drug pressure (primarily from chloroquine) was limited [28]. However, in two studies culture of polyclonal Ugandan field isolates with mixed *pfmdr1* genotypes showed, surprisingly, superior growth of the mutant 86Y allele in culture over time [29, 30]. Based on these results, it was hypothesized that mutant 86Y parasites are more fit than wild type due to epistasis with other mutations in the PfMDR1 gene. To test this hypothesis, isogenic lines engineered to contain known African PfMDR1 haplotypes in co-culture experiments were utilized, allowing direct evaluation of the impact of PfMDR1 mutations on parasite fitness, with fitness assessed by competitive in vitro growth.

## Methods

### *Plasmodium falciparum* culture

*Plasmodium falciparum* NF10 parasites with introduced PfMDR1 N86Y and Y184F mutations [14] (generously provided by M. Isabel Veiga and David Fidock, Columbia University) were cultured in complete medium consisting of RPMI-1640 (Invitrogen) supplemented with 0.5% Albumax II (GIBCO Life Technologies), 0.2% sodium bicarbonate, 100 µM hypoxanthine, 2 mM l-glutamine, 5 ug/mL gentamycin, and 25 mM HEPES at 2% haematocrit at 37 °C in an atmosphere of 5% O_2_, 5% CO_2_, and 90% N_2_.

### Growth competition experiments

Parasites were synchronized at the ring stage using 5% (wt/vol) D-sorbitol (Sigma-Aldrich) [31] and washed three times with RPMI medium. Infected erythrocytes were stained with SYTO-16 (Thermo Fisher Scientific), and counts were obtained with flow cytometry. Co-cultures (2.5 mL) were prepared by mixing two lines at equal parasitaemia at an initial total parasitaemia of 0.5% in the culture conditions noted above. Media was changed and dilutions made to maintain a parasitaemia of ~ 0.5% every 2 days. For each combination studied, 2–6 independent experiments were maintained for 32 to 40 days.

### Co-culture with anti-malarials

Parasites were cultured with varied concentrations of chloroquine (Sigma-Aldrich), lumefantrine (a gift from the Medicines for Malaria Venture), and piperaquine (Jinan Jiaquan International Trade Co.), both with continuous and intermittent exposure to the drugs, to identify maximum concentrations that allowed growth in culture. Parasites were then co-cultured in the indicated concentrations of the anti-malarials, with cycles of 2 days with and 2 days without the drugs, and culture conditions and maintenance of parasitaemia at ~ 0.5%, as described above.

### Genetic analysis of cultured parasites

Culture aliquots were collected and stored as dried blood spots on Whatman 3 MM filter paper every 4 days. DNA was extracted with Chelex 100, as previously described [32]. The ratios of co-cultured lines was determined by pyrosequencing of extracted DNA samples by EpigenDx (Hopkinton, MA), following methods as previously described [30]. In brief, biotinylated PCR products were bound to streptavidin beads and converted to single-stranded DNA sequencing templates, and the single-stranded DNA was sequenced with previously published, position-specific primers [30] using a pyrosequencing PSQ96 HS system following the manufacturer’s instructions (Qiagen). Every pyrosequencing run included negative and positive controls with known sequence; as pyrosequencing data are provided within a sequence context, the likelihood of false reads is low. The sequences of each sample were analysed using PSQ software, and alleles were quantified as the percentage of each base at sites of interest.

### Statistical analysis

The ratio of the percentage of each base at sites of interest for each pyrosequenced sample was natural log-transformed and used to calculate the fitness (*ω’*) of the mutant allele as per the relationship, $\text{ln}\left(\frac{{p}_{t}}{{q}_{t}}\right)=\text{ln}\left(\frac{{p}_{0}}{{q}_{0}}\right)+tln\left(\omega \right)$. The relative fitness index (*ω’*) for the mutant variant in each competition assay was determined from the slope of the least-squared regression plot of log(*p*_*t*_ /*q*_*t*_) against *t*, where *t* is time measured as asexual generations (2 days), *p*_*t*_ is the frequency of the wild-type variant at each generation, and *q*_*t*_ is the frequency of the mutant variant [33]. Data from multiple competition assays were utilized in fitting this linear relationship. Selection coefficients were then derived from *ω’* as per the relationship *s* = *ω’* – 1, such that *s* > 0 indicates inferior growth of the mutant and *s* < 0 indicates superior growth of the mutant compared to that of the wild type. Statistical significance was assessed using RStudio [34]. A one-way ANOVA test was used to determine the statistical significance of *s* differing from 0, and a two-way ANOVA with Tukey’s post-hoc test to compare *s* in the presence or absence of drug. *P*-values < 0.05 were considered significant.

### CRISPR-Cas9-mediated editing of *pfmdr1*

Editing was performed based on a strategy and methods described previously [35]. Plasmids pCas9-gRNA-h*dhfr* and pT7Pol-*bsd* containing gRNA8, a guide RNA that was previously demonstrated to be effective in editing other nucleotides in *pfmdr1* [35] and is located 481 nucleotides downstream of the target, were kindly provided by Caroline L. Ng and David Fidock, Columbia University. These express human dihydrofolate reductase (*dhfr*) and blasticidin S-deaminase selectable markers, respectively. A 1.4 kb fragment of donor template encoding mutant (1246Y) PfMDR1 was amplified from genomic DNA using 5′-GAGCTCGTATTTGCTGTAAGAGCTAGATTAA-3′ (SacI restriction site underlined) and 5′-GACGTCTTTAGCTAATTTTACATATTTTTTATATATTCC-3′ (AatII restriction site underlined). The Q5 Site‐Directed Mutagenesis Kit (NEB) and primers p4358 (5′-CAAGTTGATGAGTTTGAAGGGAGATTCAGAAAATGCAAAATTATC-3′) and p4359 (5′-CCAGCATAACTACCAGTAAATATAAAAGTAAATAAG-3′) were used to introduce silent mutations at the Cas9 binding site to prevent Cas9 cleavage of the plasmid or the edited gene. The resulting segment was then inserted into the pT7Pol‐*bsd* plasmid using the SacI and AatII restriction sites. Synchronized ring stage NF10 parasites at 5% parasitaemia were electroporated with 50–100 µg pCas9-gRNA-h*dhfr* and pT7Pol *bsd* plasmids in 800 µL cytomix according to previously published methods [35]. Blasticidin S HCl (2 µg/mL; Santa Cruz Biotechnology) and WR99210 (2.5 nM; Sigma-Aldrich) were supplemented 24 h post-electroporation for 6 days, followed by culture in drug free media. By day 21–28, parasites were seen on smears, parasite DNA was Sanger sequenced (Eurofin Genomics), and parasites were cloned via limiting dilution to 0.5 parasites per well in 96-well plates for a total volume of 200 µL. Media was changed weekly, and thick smears were prepared on day 14 and then weekly. Wells positive for parasites were expanded to 500 µL cultures in 24-well plates, and DNA was extracted and sequenced using primers 1246 F (5′- ATGATCACATTATATTAAAAAATGATATGACAAAT-3′) and MDR-O2 (5′- ATGATTCGATAAATTCATCTATAGCAGCAA-3′). Sequencing results were compared to the sequence of *pfmdr1* (PF3D7_0523000) for the reference 3D7 strain.

## Results

### Parasites under study

**Fig. 1:**
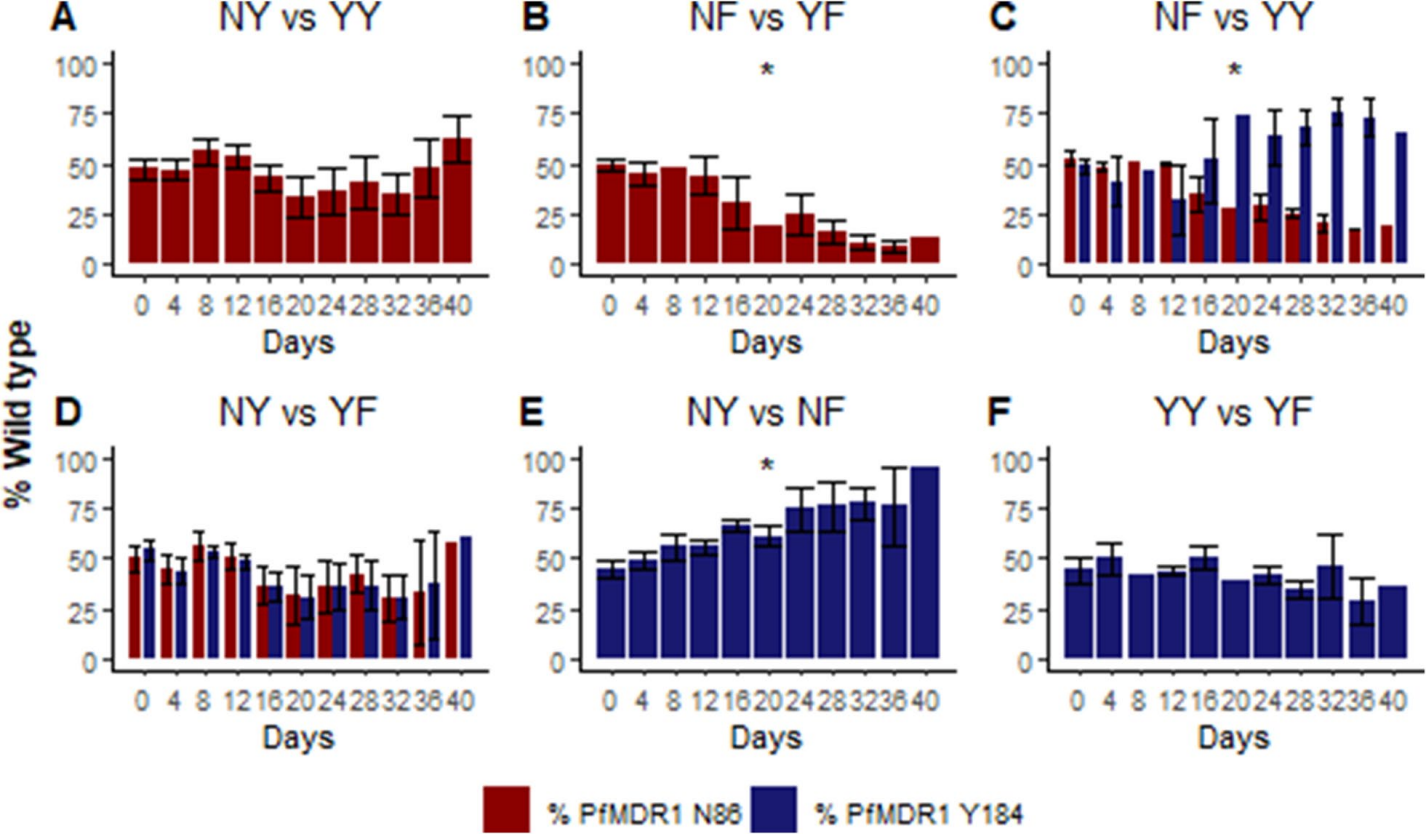
Relative fitness of parasites with varied PfMDR1 sequences. Combinations of PfMDR1 N86Y and Y184F haplotypes were co-cultured in competitive growth experiments, beginning with equal quantities of the two lines, and relative fitness was assessed based on the prevalence of each genotype over 40 days, with the percentage of wild type shown for the loci that differed between the two lines. Each line is designated based on the amino acid at positions 86 and 184. Co-culture experiments demonstrated three lines in equipoise (**A**, **D**, **F**), overgrowth of mutant parasites (**B**), overgrowth of wild-type parasites (**E**), and selection of wild type/mixed 86Y/Y184 parasites (**C**). * Indicates combinations with statistically significant selection coefficients

*Plasmodium falciparum* strain NF10, the product of a genetic cross between the Brazilian 7G8 and Ghanaian GB4 strains, was engineered previously to provide lines wild type or mutant at PfMDR1 N86Y and Y184F, loci that have been polymorphic in African parasites [14] (Table 1). Considering other common polymorphisms relevant to drug resistance, NF10 parasites have wild type (based on strain 3D7) sequence at PfMDR1 positions 1034, 1042, and 1246; have one copy of the *pfmdr1* gene; and contain the PfCRT position 72–76 CVIET haplotype and 5 other PfCRT mutations (A220S, Q271E, N326S, I356T, and R371I), a genotype associated with resistance to chloroquine [36]. Lines with all possible combinations of PfMDR1 N86Y and Y184F were subjected to competition experiments, in which equal quantities of two lines were co-cultured for 40 days, and the proportion of each line was quantified by pyrosequencing every 4 days (Fig. 1). Relative growth estimates were characterized based on the ability of a line to outgrow the co-cultured line over 40 days, and used to derive per-generation relative selection coefficients (*s*) for wild type alleles in each competitive growth experiment (Table 2).

**Table 1:** NF10 haplotypes studied in competitive growth experiments

Haplotype	PfMDR1 sequence		Drug sensitivity (IC_50_, nM)		
	86	184	Chloroquine	Lumefantrine	Piperaquine
NY	Wild type	Wild type	185	3.2	28.9
NF	Wild type	Mutant	205	3.2	35.1
YY	Mutant	Wild type	331	0.9	35.4
YF	Mutant	Mutant	328	0.9	40.2

The haplotypes shown indicate the N86Y and Y184F sequences. Drug sensitivities are as reported previously [14]

**Table 2:** In vitro growth selection coefficients for co-cultured PfMDR1 variants

Haplotype combination	N	PfMDR1 N86		PfMDR1 Y184	
		*s*	Pr(>|t|)	*s*	Pr(>|t|)
NY vs. YY	5	− 0.02	0.39	–	–
NF vs. YF	3	− 0.12	< 0.0001	–	–
NF vs. YY	3	− 0.09	< 0.0001	0.09	0.008
NY vs. YF	4	− 0.04	0.07	− 0.04	0.06
NY vs. NF	4	–	–	0.13	< 0.0001
YY vs. YF	3	–	–	− 0.03	0.17

The haplotypes shown indicate the N86Y and Y184F sequences. The per-generation selection coefficient (*s*) was derived from the relative fitness index (*ω’*) as per the relationship *s* = *ω’* – 1, such that *s* < 0 and *s* > 0 indicate inferior and superior growth of the wild-type allele, respectively. *s* was considered to differ significantly from 0 when Pr(>|t|) was ≤ 0.05. N, number of independent growth competition assays analysed

### Impact of N86Y on parasite fitness

With the wild type Y184 sequence fixed, parasites wild type or mutant at position 86 were in equipoise (Fig. 1A, Table 2). With the mutant 184 F sequence fixed, parasites mutant at position 86 overgrew wild type parasites (Fig. 1B; Table 2). Considering lines differing at both positions, 86Y/Y184 parasites overgrew N86/184F parasites (Fig. 1C, Table 2) and N86/Y184 and 86Y/184F parasites were in equipoise (Fig. 1D; Table 2). Taken together, these results suggest that the N86Y polymorphism has limited impact on parasite fitness when the 184 sequence is wild type, but that in the setting of the 184 F mutation, surprisingly, mutant 86Y parasites are more fit than those wild type at position 86.

### Impact of Y184F on parasite fitness

With the wild type N86 sequence fixed, parasites wild type at position 184 overgrew those mutant at this position (Fig. 1E; Table 2). With the mutant 86Y sequence fixed, parasites wild type or mutant at position 184 were in equipoise (Fig. 1F, Table 2). Taken together, these results suggest that in the setting of the N86 wild type sequence, parasites with the Y184 wild type polymorphism are more fit than those with a mutation at this position.

### Impact of D1246Y on parasite fitness

**Fig. 2:**
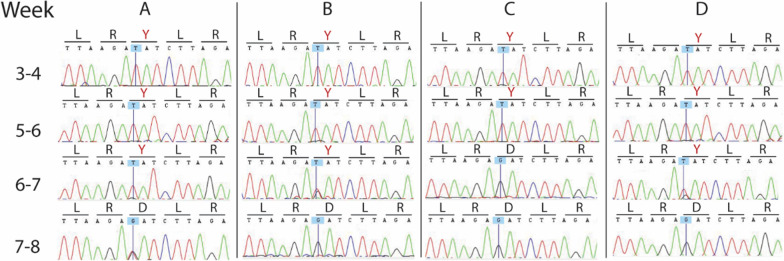
Transfection of PfMDR1 1246Y over time. Sequence traces for 4 independent transfections are displayed (**A–D**). Sequencing was conducted 3–4 weeks post-transfection and then weekly during cloning and expansion. The sequences shown are for amino acids 1244 to 1248. Nucleotides highlighted in blue and with a vertical line represent those subject to the T to G substitution that corresponds to a tyrosine (Y, mutant) to aspartic acid (**D**, wild type) substitution

NF10 parasites engineered to carry the PfMDR1 1246Y mutation were previously unavailable. Engineering the 1246Y mutation into NF10 parasites with and without mutations at N86Y and Y184F was attempted using the CRISPR Cas9 system, with 6 days of selective drugs to maintain transfected plasmids followed by culture in drug-free media. By day 21–28, parasites were seen on smears and cloned by limiting dilution, and parasite DNA was isolated and sequenced weekly. In multiple experiments, despite successful integration, parasites with 1246Y were not stable in culture. Consistently, the mutant sequence was replaced by wild type over time (Fig. 2). Presumably, despite limiting dilution these cultures contained > 1 parasite clone, allowing overgrowth over time of more fit wild type parasites that were initially at too low abundance to be recognized by sequencing. As numerous other diluted cultures contained no parasites, but none had persistence of mutant parasites, it appears that the 1246Y mutation incurs a substantial fitness cost, regardless of N86Y/Y184F haplotype, when introduced in an NF10 background.

### Impacts of anti-malarials on parasite fitness

**Fig. 3:**
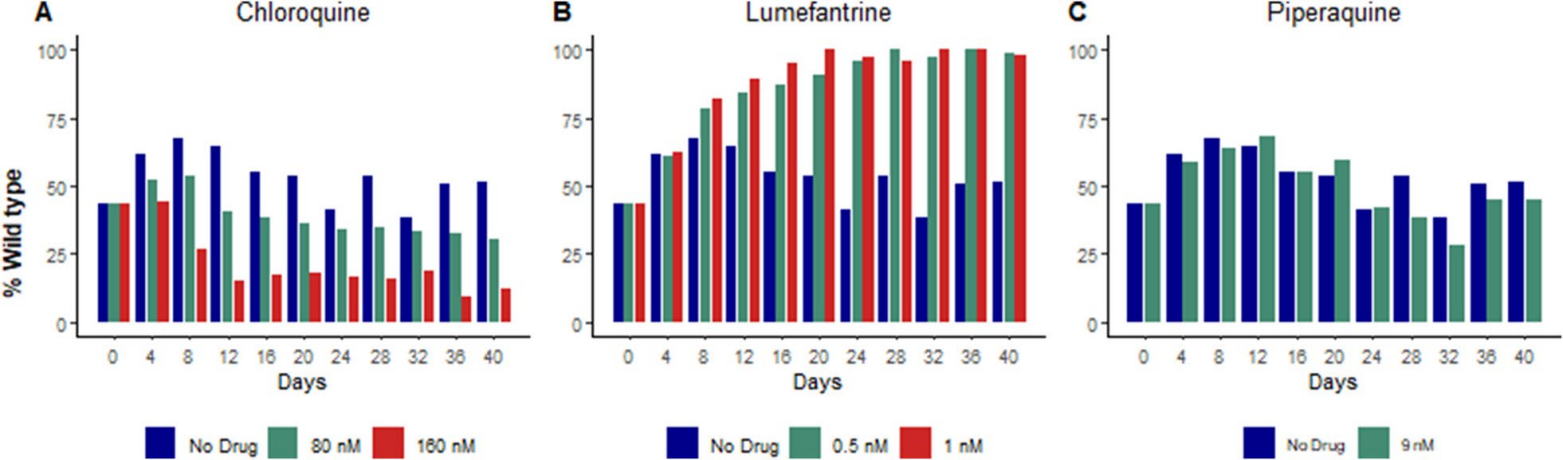
Impact of anti-malarials on fitness of parasites with varied PfMDR1 sequences. With wild-type fixed at PfMDR1 184, parasites wild-type or mutant at 86 (NY vs. YY) were co-cultured in the presence of the indicated concentrations of chloroquine (**A**), lumefantrine (**B**), or piperaquine (**C**) and the prevalence of each genotype was followed over 36 days. It is seen that mutant parasites are favored under chloroquine pressure, wild type parasites are favored under lumefantrine pressure, and that there is no clear impact on genotype prevalence under piperaquine pressure

Co-culture experiments considering different PfMDR1 86 sequences (N86/Y184 vs. 86Y/Y184 parasites) were performed in the presence of selective concentrations (50 and 25% IC_50_ for chloroquine and lumefantrine and 25% IC_50_ for piperaquine) of anti-malarials. During co-culture with chloroquine, parasites with the 86Y mutation overgrew those wild type at this locus (Fig. 3; Table 3). During co-culture with lumefantrine, wild type N86 parasites overgrew mutant parasites. During co-culture with piperaquine, mutant and wild type parasites were in equilibrium. Overall, anti-malarials modified the impacts on fitness of the N86Y polymorphism in a manner consistent with selections seen in clinical studies (Table 4).

**Table 3:** In vitro growth selection coefficients for PfMDR1 variants (NY vs. YY) co-cultured in the presence of anti-malarials

Drug exposure	*s*	*P*_*s*_	P_*d*_
No drug	− 0.02	0.20	-
Chloroquine			
80 nM, 25% IC_50_	− 0.04	0.0006	0.67
160 nM, 50% IC_50_	− 0.08	0.001	0.03
Lumefantrine			
0.5 nM, 25% IC_50_	0.49	0.003	< 0.0001
1 nM, 50% IC_50_	0.43	0.0008	< 0.0001
Piperaquine			
9 nM, 25% IC_50_	− 0.05	0.08	0.63

Analysis was as described for Table 1. *P*_*s*_, *P* value for comparison of *s* to 0. *P*_*d*,_ *P* value for comparison of *s* for assays performed in the presence or absence of anti-malarials. *P*_*s*_ was determined using one-way ANOVA. *P*_*d*_ was determined using two-way ANOVA with Tukey’s post-hoc test

**Table 4:** Summary of selection results

Selective pressure	Selected against	Selected for
None	NF	NY, YF, YY
Chloroquine	NY	YY
Lumefantrine	YY	NY
Piperaquine	No selection	

The haplotypes shown indicate the N86Y and Y184F sequences

## Discussion

The in vitro fitness of *P. falciparum* lines with different PfMDR1 genetic polymorphisms that have been linked to sensitivity to key anti-malarial drugs was compared [12, 13]. Surprisingly, the PfMDR1 86Y mutation, which mediates decreased sensitivity to chloroquine and amodiaquine independent of mutations in *pfcrt* [10, 14], but increased sensitivity to lumefantrine and mefloquine, was associated with enhanced fitness compared to wild type, but only in the presence of the 184F mutation. Likewise, the wild type Y184 sequence was favored, except in the presence of the 86Y mutation. A third PfMDR1 mutation that has been common in parts of Africa, 1246Y, was unstable in culture. Culture with anti-malarials stabilized polymorphisms previously associated with decreased sensitivity to those drugs. Taken together, these results suggest a complex interplay between PfMDR1 mutations and parasite fitness, consistent with the changes in anti-malarial therapy and *P. falciparum* genotypes seen in Africa in recent years.

*Plasmodium falciparum* genotypes associated with sensitivity to key ACT partner drugs have changed remarkably in Africa over the last decade. Notably, the prevalence of two polymorphisms that are associated with decreased sensitivity to chloroquine and amodiaquine, PfCRT 76T and PfMDR1 86Y, has decreased dramatically [11]. These changes have followed widespread adoption of ACT, in particular with artemether-lumefantrine, to treat uncomplicated malaria. Thus, it is reasonable to ascribe changes in genotypes to changes in drug pressure. However, some common polymorphisms have unclear associations with drug sensitivity, and so their prevalence might be better explained by impacts on fitness. This seems most likely for Y184F, which was not clearly associated with ex vivo drug sensitivity [10], and which maintains an intermediate prevalence in much of Africa. This study suggests that, in the context of heavy use of chloroquine, the 184F mutation persisted due to fitness advantages of the YF haplotype over the NF haplotype. More recently, with reversion to N86 wild type parasites, likely due to decreased selective pressure from chloroquine and increased pressure from lumefantrine, it is reasonable to expect that the 184F mutation would decrease in prevalence, but in Uganda the prevalence of this polymorphism has remained fairly stable in recent years [37, 38]. Fitness impacts of PfMDR1 polymorphisms should be considered in light of other polymorphisms that have also been shown to affect drug sensitivity and/or fitness, most notably the PfMDR1 D1246Y and PfCRT 76T mutations [39], but also other PfCRT mutations recently identified in southeast Asian parasites [40, 41], and mediators of artemisinin resistance in southeast Asia [42–44]. The inability to produce parasites with a stable PfMDR1 1246Y mutation capable of in vitro growth was highly suggestive of diminished fitness for mutant parasites, but this finding prevented comparison of fitness using competitive growth experiments.

It is important to put these results in a clinical context, considering widespread treatment of malaria with ACT (primarily artemether-lumefantrine) in Africa. Clinical trials have shown continued excellent efficacy for leading artemisinin-based combinations across Africa [45–47]. However, in Uganda relative loss of efficacy of artemether-lumefantrine compared to artesunate-amodiaquine was seen, consistent with the genotypic changes seen in recent years [45]. These new results suggest that the PfMDR1 86Y chloroquine/amodiaquine resistance mutation is stabilized in the presence of specific PfMDR1 haplotypes, but that drug pressure strongly influences selection of parasite genotypes. As noted above, with widespread utilization of artemether-lumefantrine, the 86Y mutation is disappearing, indicating selective pressure in the field consistent with that seen in these co-culture experiments, and modestly decreasing the anti-malarial efficacy of artemether-lumefantrine [45].

This study had important limitations. Although comparison of isogenic lines differing only at introduced loci provides assurance that the impacts of the introduced polymorphisms were compared, relative growth in culture may not be representative of relative fitness during human infections. The studied parasites were the products of a cross between African and Brazilian clones; arguably consideration of African parasites may have yielded results more relevant for drug resistance in Africa. It was not possible to study all PfMDR1 mutations commonly circulating in African parasites, as stable parasites with the PfMDR1 1246Y mutation could not be produced. The whole genome sequences of transfected parasites were not compared; it is possible, though unlikely, that additional mutations that occurred by chance during the transfection process may have impacted on parasite fitness. Most importantly, multiple factors impact upon parasite survival in clinical infections, including inherent parasite fitness, host immunity, multiplicity of infection, drug pressure over the course of infection, and host genetics. In this context, these experiments have limited ability to predict the haplotypes that will emerge and become stable over time, but it is of interest that these results are generally consistent with the patterns of drug use and haplotype prevalence in Africa. However, while it was demonstrated that the PfMDR1 184F mutation stabilizes the 86Y mutation, these data cannot explain the persistence of 184F as 86Y has disappeared in Uganda in recent years. Deeper understanding of responses to additional selective environments will be necessary to better understand and predict evolutionary outcomes.

## Conclusions

In summary, varied impacts on fitness of PfMDR1 polymorphisms were associated with altered drug sensitivity. Notably, the 86 N wild type that mediates decreased sensitivity to lumefantrine had decreased fitness in the setting of the 184 F mutation compared to the Y184 wild type. An appreciation of the contributions of different polymorphisms and haplotypes to parasite fitness can help us to predict changes in parasites under the pressure of anti-malarial drug use, and thus to best utilize available drugs to treat and control malaria.

## Data Availability

All data and materials from this study are available upon request to the corresponding author.

## References

[1] WHO (2019). World malaria report, 2019.

[2] Ashley EA, Pyae Phyo A, Woodrow CJ, Malaria (2018). Lancet.

[3] Nosten F, White NJ (2007). Artemisinin-based combination treatment of falciparum malaria. Am J Trop Med Hyg.

[4] WHO (2015). Guidelines for the treatment of malaria.

[5] Ashley EA, Dhorda M, Fairhurst RM, Amaratunga C, Lim P, Suon S, Sreng S, Anderson JM, Mao S, Sam B (2014). Spread of artemisinin resistance in *Plasmodium falciparum* malaria. N Engl J Med.

[6] Cui L, Mharakurwa S, Ndiaye D, Rathod PK, Rosenthal PJ (2015). Antimalarial drug resistance: literature review and activities and findings of the ICEMR network. Am J Trop Med Hyg.

[7] Rosenthal PJ (2013). The interplay between drug resistance and fitness in malaria parasites. Mol Microbiol.

[8] Valderramos SG, Fidock DA (2006). Transporters involved in resistance to antimalarial drugs. Trends Pharmacol Sci.

[9] Eyase FL, Akala HM, Ingasia L, Cheruiyot A, Omondi A, Okudo C (2013). The role of Pfmdr1 and Pfcrt in changing chloroquine, amodiaquine, mefloquine and lumefantrine susceptibility in Western-Kenya *P. falciparum* samples during 2008–2011. PLoS ONE.

[10] Rasmussen SA, Ceja FG, Conrad MD, Tumwebaze PK, Byaruhanga O, Katairo T (2017). Changing antimalarial drug sensitivities in Uganda. Antimicrob Agents Chemother.

[11] Conrad MD, Rosenthal PJ (2019). Antimalarial drug resistance in Africa: the calm before the storm?. Lancet Infect Dis.

[12] Koenderink JB, Kavishe RA, Rijpma SR, Russel FG (2010). The ABCs of multidrug resistance in malaria. Trends Parasitol.

[13] Gil JP, Krishna S (2017). pfmdr1 (*Plasmodium falciparum* multidrug drug resistance gene 1): a pivotal factor in malaria resistance to artemisinin combination therapies. Expert Rev Anti Infect Ther.

[14] Veiga MI, Dhingra SK, Henrich PP, Straimer J, Gnadig N, Uhlemann AC (2016). Globally prevalent PfMDR1 mutations modulate *Plasmodium falciparum* susceptibility to artemisinin-based combination therapies. Nat Commun.

[15] Nsobya SL, Dokomajilar C, Joloba M, Dorsey G, Rosenthal PJ (2007). Resistance-mediating *Plasmodium falciparum* pfcrt and pfmdr1 alleles after treatment with artesunate-amodiaquine in Uganda. Antimicrob Agents Chemother.

[16] Humphreys GS, Merinopoulos I, Ahmed J, Whitty CJ, Mutabingwa TK, Sutherland CJ (2007). Amodiaquine and artemether-lumefantrine select distinct alleles of the *Plasmodium falciparum* mdr1 gene in Tanzanian children treated for uncomplicated malaria. Antimicrob Agents Chemother.

[17] Zongo I, Dorsey G, Rouamba N, Tinto H, Dokomajilar C, Guiguemde RT (2007). Artemether-lumefantrine versus amodiaquine plus sulfadoxine-pyrimethamine for uncomplicated falciparum malaria in Burkina Faso: a randomised non-inferiority trial. Lancet.

[18] Sisowath C, Stromberg J, Martensson A, Msellem M, Obondo C, Bjorkman A (2005). In vivo selection of *Plasmodium falciparum* pfmdr1 86 N coding alleles by artemether-lumefantrine (Coartem). J Infect Dis.

[19] Some AF, Sere YY, Dokomajilar C, Zongo I, Rouamba N, Greenhouse B (2010). Selection of known *Plasmodium falciparum* resistance-mediating polymorphisms by artemether-lumefantrine and amodiaquine-sulfadoxine-pyrimethamine but not dihydroartemisinin-piperaquine in Burkina Faso. Antimicrob Agents Chemother.

[20] Froberg G, Ferreira PE, Martensson A, Ali A, Bjorkman A, Gil JP (2013). Assessing the cost-benefit effect of a *Plasmodium falciparum* drug resistance mutation on parasite growth in vitro. Antimicrob Agents Chemother.

[21] Price RN, Uhlemann AC, Brockman A, McGready R, Ashley E, Phaipun L (2004). Mefloquine resistance in *Plasmodium falciparum* and increased pfmdr1 gene copy number. Lancet.

[22] Mungthin M, Khositnithikul R, Sitthichot N, Suwandittakul N, Wattanaveeradej V, Ward SA (2010). Association between the pfmdr1 gene and in vitro artemether and lumefantrine sensitivity in Thai isolates of *Plasmodium falciparum*. Am J Trop Med Hyg.

[23] Holmgren G, Bjorkman A, Gil JP (2006). Amodiaquine resistance is not related to rare findings of pfmdr1 gene amplifications in Kenya. Trop Med Int Health.

[24] Witkowski B, Nicolau ML, Soh PN, Iriart X, Menard S, Alvarez M (2010). *Plasmodium falciparum* isolates with increased pfmdr1 copy number circulate in West Africa. Antimicrob Agents Chemother.

[25] Conrad MD, LeClair N, Arinaitwe E, Wanzira H, Kakuru A, Bigira V (2014). Comparative impacts over 5 years of artemisinin-based combination therapies on *Plasmodium falciparum* polymorphisms that modulate drug sensitivity in Ugandan children. J Infect Dis.

[26] Hayward R, Saliba KJ, Kirk K (2005). pfmdr1 mutations associated with chloroquine resistance incur a fitness cost in *Plasmodium falciparum*. Mol Microbiol.

[27] Preechapornkul P, Imwong M, Chotivanich K, Pongtavornpinyo W, Dondorp AM, Day NP (2009). *Plasmodium falciparum* pfmdr1 amplification, mefloquine resistance, and parasite fitness. Antimicrob Agents Chemother.

[28] Ord R, Alexander N, Dunyo S, Hallett R, Jawara M, Targett G (2007). Seasonal carriage of pfcrt and pfmdr1 alleles in Gambian *Plasmodium falciparum* imply reduced fitness of chloroquine-resistant parasites. J Infect Dis.

[29] Nsobya SL, Kiggundu M, Joloba M, Dorsey G, Rosenthal PJ (2008). Complexity of *Plasmodium falciparum* clinical samples from Uganda during short-term culture. J Infect Dis.

[30] Ochong E, Tumwebaze PK, Byaruhanga O, Greenhouse B, Rosenthal PJ (2013). Fitness consequences of *Plasmodium falciparum* pfmdr1 polymorphisms inferred from ex vivo culture of Ugandan parasites. Antimicrob Agents Chemother.

[31] Lambros C, Vanderberg JP (1979). Synchronization of *Plasmodium falciparum* erythrocytic stages in culture. J Parasitol.

[32] Plowe CV, Djimde A, Bouare M, Doumbo O, Wellems TE (1995). Pyrimethamine and proguanil resistance-conferring mutations in *Plasmodium falciparum* dihydrofolate reductase: polymerase chain reaction methods for surveillance in Africa. Am J Trop Med Hyg.

[33] Gabryszewski SJ, Dhingra SK, Combrinck JM, Lewis IA, Callaghan PS, Hassett MR (2016). Evolution of fitness cost-neutral mutant PfCRT conferring *P. falciparum* 4-aminoquinoline drug resistance is accompanied by altered parasite metabolism and digestive vacuole physiology. PLoS Pathog.

[34] RStudio Team (2020). RStudio: Integrated development for R.

[35] Ng CL, Siciliano G, Lee MC, de Almeida MJ, Corey VC, Bopp SE (2016). CRISPR-Cas9-modified pfmdr1 protects *Plasmodium falciparum* asexual blood stages and gametocytes against a class of piperazine-containing compounds but potentiates artemisinin-based combination therapy partner drugs. Mol Microbiol.

[36] Sa JM, Twu O, Hayton K, Reyes S, Fay MP, Ringwald P (2009). Geographic patterns of *Plasmodium falciparum* drug resistance distinguished by differential responses to amodiaquine and chloroquine. Proc Natl Acad Sci USA.

[37] Tumwebaze P, Tukwasibwe S, Taylor A, Conrad M, Ruhamyankaka E, Asua V (2017). Changing antimalarial drug resistance patterns identified by surveillance at three sites in Uganda. J Infect Dis.

[38] Asua V, Vinden J, Conrad MD, Legac J, Kigozi SP, Kamya MR (2019). Changing molecular markers of antimalarial drug sensitivity across Uganda. Antimicrob Agents Chemother.

[39] Djimde A, Doumbo OK, Cortese JF, Kayentao K, Doumbo S, Diourte Y (2001). A molecular marker for chloroquine-resistant falciparum malaria. N Engl J Med.

[40] Dhingra SK, Gabryszewski SJ, Small-Saunders JL, Yeo T, Henrich PP, Mok S (2019). Global spread of mutant PfCRT and its pleiotropic impact on *Plasmodium falciparum* multidrug resistance and fitness. MBio.

[41] Ross LS, Dhingra SK, Mok S, Yeo T, Wicht KJ, Kumpornsin K (2018). Emerging Southeast Asian PfCRT mutations confer *Plasmodium falciparum* resistance to the first-line antimalarial piperaquine. Nat Commun.

[42] Straimer J, Gnadig NF, Stokes BH, Ehrenberger M, Crane AA, Fidock DA (2017). *Plasmodium falciparum* K13 mutations differentially impact ozonide susceptibility and parasite fitness in vitro. mBio.

[43] Nair S, Li X, Arya GA, McDew-White M, Ferrari M, Nosten F (2018). Fitness Costs and the rapid spread of kelch13-C580Y substitutions conferring artemisinin resistance. Antimicrob Agents Chemother.

[44] Tirrell AR, Vendrely KM, Checkley LA, Davis SZ, McDew-White M, Cheeseman IH (2019). Pairwise growth competitions identify relative fitness relationships among artemisinin resistant *Plasmodium falciparum* field isolates. Malar J.

[45] Yeka A, Kigozi R, Conrad MD, Lugemwa M, Okui P, Katureebe C (2016). Artesunate/amodiaquine versus artemether/lumefantrine for the treatment of uncomplicated malaria in Uganda: a randomized trial. J Infect Dis.

[46] West African Network for Clinical Trials of Antimalarial Drugs (WANECAM) (2018). Pyronaridine-artesunate or dihydroartemisinin-piperaquine versus current first-line therapies for repeated treatment of uncomplicated malaria: a randomised, multicentre, open-label, longitudinal, controlled, phase 3b/4 trial. Lancet.

[47] Yeka A, Wallender E, Mulebeke R, Kibuuka A, Kigozi R, Bosco A (2019). Comparative efficacy of artemether-lumefantrine and dihydroartemisinin-piperaquine for the treatment of uncomplicated malaria in Ugandan children. J Infect Dis.

